# Elucidation of Novel Therapeutic Targets for Acute Myeloid Leukemias with *RUNX1*-*RUNX1T1* Fusion

**DOI:** 10.3390/ijms20071717

**Published:** 2019-04-06

**Authors:** Jae Won Yun, Yoon Kyung Bae, So Yeong Cho, Harim Koo, Hee-Jin Kim, Do-Hyun Nam, Sun-Hee Kim, Sejong Chun, Kyeung Min Joo, Woong-Yang Park

**Affiliations:** 1 Department of Health Sciences and Technology, Samsung Advanced Institute for Health Science and Technology (SAIHST), Sungkyunkwan University, Seoul 06351, Korea; jwyunmd@gmail.com (J.W.Y.); bab1825@gmail.com (Y.K.B.); guhalim@naver.com (H.K.); dhns.nam@samsung.com (D.-H.N.); 2 Department of Laboratory Medicine & Genetics, Samsung Medical Center, Sungkyunkwan University School of Medicine, Seoul 06351, Korea; heejinkim@skku.edu (H.-J.K.); sunnyhk@skku.edu (S.-H.K.); 3 Single Cell Network Research Center, Sungkyunkwan University of Medicine, Suwon 16419, Korea; soyeong159@naver.com; 4 Department of Anatomy and Cell biology, Sungkyunkwan University School of Medicine, Suwon 16419, Korea; 5 Departments of Neurosurgery, Samsung Medical Center, Sungkyunkwan University School of Medicine, Seoul 06351, Korea; 6 Department of Laboratory Medicine, Chonnam National University Medical School & Hospital, Gwangju 61469, Korea; 7 Samsung Genome Institute, Samsung Medical Center, Seoul 06351, Korea; 8 Departments of Molecular Cell Biology, Sungkyunkwan University School of Medicine, Suwon 16419, Korea

**Keywords:** acute myeloid leukemia, *RUNX1-RUNX1T1*, oncogenes, transcription factor

## Abstract

The *RUNX1-RUNX1T1* fusion is a frequent chromosomal alteration in acute myeloid leukemias (AMLs). Although *RUNX1-RUNX1T1* fusion protein has pivotal roles in the development of AMLs with the fusion, *RUNX1-RUNX1T1*, fusion protein is difficult to target, as it lacks kinase activities. Here, we used bioinformatic tools to elucidate targetable signaling pathways in AMLs with *RUNX1-RUNX1T1* fusion. After analysis of 93 AML cases from The Cancer Genome Atlas (TCGA) database, we found expression of 293 genes that correlated to the expression of the *RUNX1-RUNX1T1* fusion gene. Based on these 293 genes, the cyclooxygenase (COX), vascular endothelial growth factor receptor (VEGFR), platelet-derived growth factor receptor (PDGFR), and fibroblast growth factor receptor (FGFR) pathways were predicted to be specifically activated in AMLs with *RUNX1-RUNX1T1* fusion. Moreover, the in vitro proliferation of AML cells with *RUNX1-RUNX1T1* fusion decreased significantly more than that of AML cells without the fusion, when the pathways were inhibited pharmacologically. The results indicate that novel targetable signaling pathways could be identified by the analysis of the gene expression features of AMLs with non-targetable genetic alterations. The elucidation of specific molecular targets for AMLs that have a specific genetic alteration would promote personalized treatment of AMLs and improve clinical outcomes.

## 1. Introduction

Acute myeloid leukemia (AML) with *RUNX1-RUNX1T1* fusion consists of up to 5% of all AML or 10% of AML M2 subtype in the French-American-British classification [1]. The *RUNX1-RUNX1T1* fusion transcript results in aberrant products that create nuclear transcriptional co-repressor complexes and suppress the expression of RUNX1 target genes [2,3,4]. Since *RUNX1T1* silencing with short hairpin RNAs has shown in vitro therapeutic effects on AML cells with the *RUNX1-RUNX1T1* fusion [5], *RUNX1T1* overexpression has been suggested as a leukemogenesis factor. Alteration in the specificity of *RUNX1* to its target sequences by the fusion might also contribute to leukemogenesis [4]. Moreover, the *RUNX1-RUNX1T1* fusion is associated with the differential clinical prognosis of AML patients [6].

Specific genomic alterations not only improve our understanding of leukemogenesis mechanisms, but also are potential therapeutic targets for targeted chemotherapies. A good example would be the inhibition of enzymatic kinase activity of BCR-ABL fusion protein in chronic myeloid leukemia (CML) with imatinib (Gleevec^®^) [7]. However, as it is relatively difficult to prevent the *RUNX1-RUNX1T1* fusion protein from mediating non-enzymatic transcriptional repression in vivo, inhibition of signaling pathways modulated in AML cells with the *RUNX1-RUNX1T1* fusion cab be considered.

Herein, we utilized the RNA-sequencing (RNA-seq) data of The Cancer Genome Atlas (TCGA) [8], and compared the gene expression profile of seven *RUNX1-RUNX1T1* fusion-positive AMLs with those of 86 AMLs that have normal karyotype, in order to systemically identify important cancer signalings and alternative druggable targets of the AMLs with the *RUNX1-RUNX1T1* fusion. The analysis revealed specific activation of signaling pathways in the AMLs with the *RUNX1-RUNX1T1* fusion, including COX, VEGF, PDGF, and FGFR1, which were pharmacologically inhibited in vitro to show the specific inhibition of the proliferation of AML cells with the *RUNX1-RUNX1T1* fusion. Through in silico and in vitro validation, our study provides evidence for repurposing molecular targeting agents approved in different types of cancers or other diseases.

## 2. Results

### 2.1. Identification of RUNX1-RUNX1T1 Fusion-Positive AMLs

**Figure 1:**
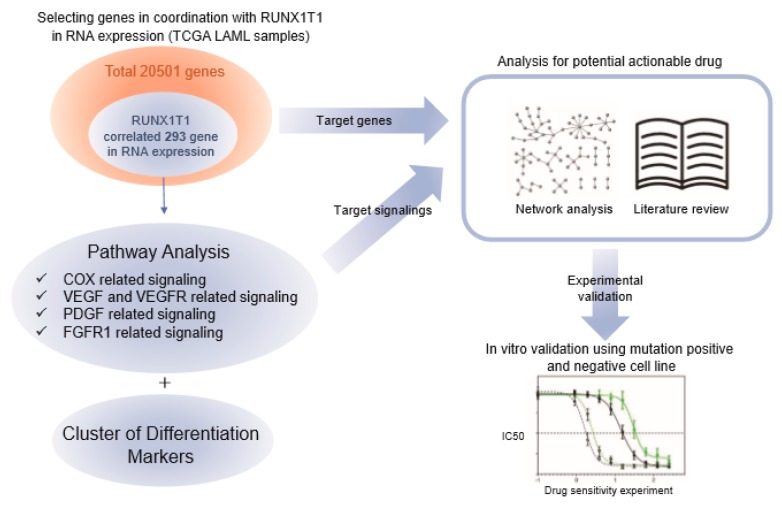
Overall scheme of this study. Transcriptome data of 200 acute myeloid leukemias (AMLs). Patients were retrieved from the Broad GDAC Firehose database. Correlated expressions with *RUNX1T1* were observed in 293 genes (upper right, red circle). Over-representation analysis results highlighted the alteration of COX, VEGF/VEGFR, PDGF, and FGFR1 related signaling pathways in sync with *RUNX1T1* expression (mid to lower right, blue circles). Analysis of potential drug reposition was performed (upper left), results of which were validated with in vitro experiment with AML cell lines (lower left).

Seven *RUNX1-RUNX1T1* fusion-positive and 86 fusion-negative (without detectable genomic structural abnormalities) AMLs were selected from 200 patients available in the Broad DAC Firehose (Figure 1).

Group-wise comparison of the clinical and pathological characteristics (Table 1) showed a difference in the mean age at diagnosis; the *RUNX1-RUNX1T1* fusion-positive patients were significantly younger than the fusion-negative patients (*p* < 0.01). In peripheral blood analysis (Table 1), the *RUNX1-RUNX1T1* fusion-positive patients had significantly lower blast counts (*p* < 0.01), hemoglobin measurements (*p* < 0.01), and platelet counts (*p* < 0.01) at diagnosis. Significantly lower bone marrow cellularity was also observed in the *RUNX1-RUNX1T1* fusion-positive AMLs (*p* < 0.01, Table 1). Although it was not exclusive, the *CD19* expression ratio of cancer cells was significantly higher in the AMLs with *RUNX1-RUNX1T1* fusion.

These clinical and pathological features of the *RUNX1-RUNX1T1* fusion-positive AMLs were well-matched with those recorded in previous reports [9,10,11,12], indicating the validity of selecting the AMLs with *RUNX1-RUNX1T1* fusion in the TCGA database.

### 2.2. Genes and Pathways Specifically Altered in AMLs with RUNX1-RUNX1T1 Fusion

Using the transcriptome data of the 7 *RUNX1-RUNX1T1* fusion-positive and 86 fusion-negative AMLs in the TCGA database, 293 genes (Figure 1 and Table S1) were identified to have expressions significantly correlated with the *RUNX1-RUNX1T1* fusion transcript level. Among the 293 genes, 42 genes were cancer-related based on the predefined cancer gene list (2027 genes, http://www.bushmanlab.org/links/genelists) (Figure S1). *CAV1*, *POU4F1*, and *ROBO1* were most significantly overexpressed in correlation with the *RUNX1T1* expression, which is concordant with the previous report [13]. Interestingly, POU4F1 is known to be up-regulated by *RUNX1-RUNX1T1* fusion, and the fusion with POU4F1 has a synergy in driving B-lymphoid gene expression in t (8:21) AML [14,15]. *FGFR1*, *VEGFA*, *NBL1*, *TET1*, *IKZF2*, *CCND1*, and *MPL* also showed positive correlations (Pearson R > 0.4) with *RUNX1T1* in RNA levels. Although not previously shown to be directly related with cancers, several surface markers, such as CD19 and CD34, showed significant positive correlation (Pearson R > 0.5) with *RUNX1T1* in RNA levels, whereas CD33 showed negative correlation (Pearson R = −0.36) (Figure S1).

**Figure 2:**
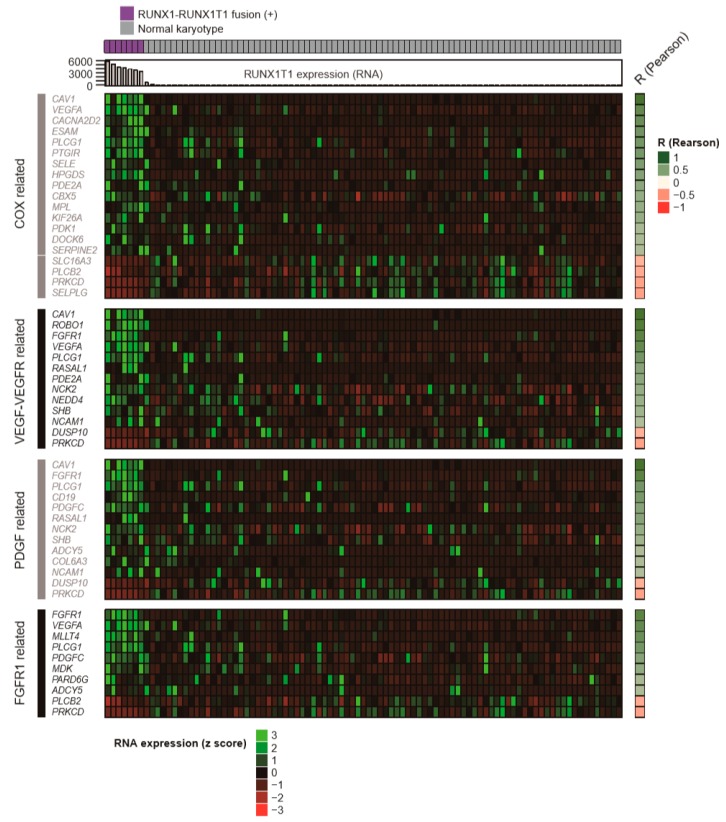
Heat Map of genes with altered expression correlating to *RUNX1T1* expression. Genes relevant to the COX, VEGF-vascular endothelial growth factor receptor (VEGFR), PDGF, and FGFR1 related pathways were found to be altered in AMLs with *RUNX1-RUNX1T1* fusion, among a total of 293 genes with over- or under-expression, compared to normal karyotype AML patients. Pathways with q value < 0.1 were selected and merged based on the pathway ontology.

Subsequently, we applied ConsensusPathDB (CPDB) to the 293 genes to elucidate the specific functional signaling pathways that are altered in the AMLs with *RUNX1-RUNX1T1* fusion (Figure 1). The analysis was performed with the 4,011 curated gene sets provided by CPDB. In the over-representation analysis (ORA) of CPDB, 24 pathways showed statistical significance (q-values < 0.1) (Table 2 and Figure 2). Gene Set Enrichment Analysis (GSEA) was utilized to cross-validate the results from ORA, using CPDB. GSEA showed good normalized enrichment scores (NESs) of over 1.5 in the VEGFR1 specific signals, the PDGFRA signaling pathway, the celecoxib pathway, and the phospholipase C mediated cascade: FGFR1 (Figure S2). The GSEA results indicated that the affected pathways were cross-validated with two independent in silico pathway analysis methods.

**Figure 3:**
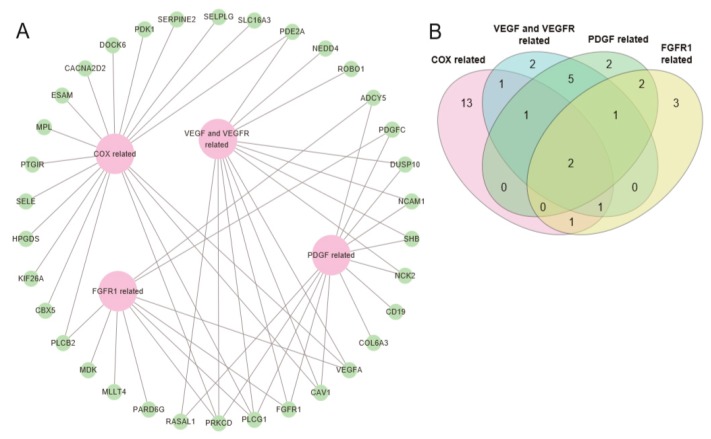
Schema of genes involved in multiple pathways. (**A**) Genes relevant to cyclooxygenase (COX), VEGF-VEGFR, PDGF, and FGFR1 related pathways are shown, and some of the genes are related to more than one pathway. (**B**) Venn diagram of the number of genes participating in each pathway. PRKCD and PLCG1 participated in all four pathways.

Several genes of the 293 genes were involved in multiple pathways in the pathway analyses. Especially, *VEGFA*, *CAV1*, *FGFR1*, *PLCG1*, and *PRKCD* were involved in at least three pathways (Figure 3), implying their functional roles in the AMLs with *RUNX1-RUNX1T1* fusion. Overall, 14 genes participated in multiple pathways (Figure 3 and Table S2).

### 2.3. Pathway Prioritization by Targeting Possibility

From the CPDB ORA results, we identified COX-2, VEGF, PDGF, and FGFR1 pathways that can be pharmacologically targeted (Table 2). The concurrent expression of *RUNX1T1* and a gene that is associated with its respective pathway are outlined in Figure 2. Overall, 34 genes were culled from the 293 genes based on their relationship with the COX-2, VEGF, PDGF, and FGFR1 pathways.

Among the 34 genes, 19 were associated with the COX-2 pathway (Figure 2). As previously suggested, *RUNX1-RUNX1T1* fusion mediates leukemogenesis through the COX pathway [16]; this result thus indicates the validity of the analysis of this study.

The VEGF/VEGFR pathway has interesting features, since 4 pathways retrieved from different database sources showed q-values < 0.1 with *p*-values < 0.004 (Table 2). This suggests the potential for drug repositioning of the VEGF/VEGFR targeting agents from anti-angiogenic treatment in order to direct AML cell-targeting therapy, which is in concordance with previous experimental studies [17]. The PDGF and FGFR1 pathways were also interesting, since they are associated with eosinophilia [18,19]. Eosinophilia has been known to be a characteristic of AMLs with *RUNX1-RUNX1T1* fusion [2]. In addition, the PDGF and FGFR1 pathways are associated with the overexpression of *CD19*, which is the immunophenotypic feature of *RUNX1-RUNX1T1* positive AMLs [2]. Considering these pathways can be pharmacologically inhibited by multiple specific targeted agents, the results suggest novel molecular targeted therapies for AMLs with *RUNX1-RUNX1T1* fusion.

### 2.4. Functional Validation of Signaling Pathways

Receptor tyrosine kinase (RTK) array was conducted to confirm the phosphorylation of the RTKs in the VEGF, PDGF, or FGFR1 pathways. The phosphorylation of 71 different RTKs was screened against SKNO-1 and Kasumi-1 human AML cells that have *RUNX1-RUNX1T1* fusion [20,21]. THP-1 AML cells were also analyzed as a negative control, as they do not harbor the *RUNX1-RUNX1T1* fusion [22]. We further analyzed the phosphorylated status of EGFRs, FGFRs, PDGFRs, and VEGFRs. EGFRs were utilized as negative controls that were not predicted in the transcriptome analyses, to be activated in AMLs with *RUNX1-RUNX1T1* fusion.

**Figure 4:**
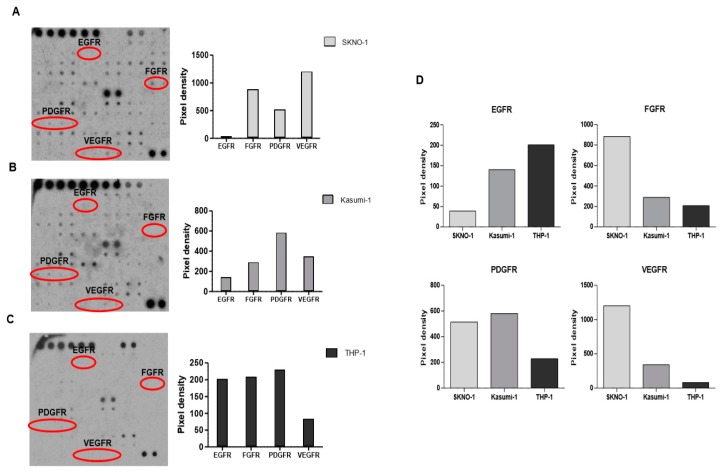
Phosphorylation status of various tyrosine kinase receptors in SKNO-1 (**A**), Kasumi-1 (**B**), and THP-1 (**C**) cells. Red circles indicate EGFR, FGFR, platelet-derived growth factor receptor (PDGFR), and VEGFR spots. (**D**) Bar graphs compare the levels of phosphorylation of EGFRs, fibroblast growth factor receptors (FGFRs), PDGFRs, and VEGFRs of the three cell lines. The three of membrane images are edited for removing background at the same level using ImageJ software.

As expected, the phosphorylation levels of FGFRs, PDGFRs, and VEGFRs were 3~5-fold higher than those of the EGFRs in both SKNO-1 (Figure 4A) and Kasumi-1 (Figure 4B). In particular, FGFR, PDGR, and VEGFR were highly phosphorylated in SKNO-1 (Figure 4A), whereas PDGFR and VEGFR were highly phosphorylated in Kasumi-1 (Figure 4B). In contrast, the phosphorylation level of EGFRs, FGFRs, and PDGFRs were almost the same in THP-1 (Figure 4C). Moreover, the phosphorylation level of the EGFRs of THP-1 were higher than those of SKNO-1 and Kasumi-1, while the phosphorylation levels of the FGFRs, PDGFRs, and VEGFRs of THP-1 were lower than those of SKNO-1 and Kasumi-1 (Figure 4D). These results indicate that the FGFR, PDGFR, and VEGFR signaling pathways may be specifically activated in AMLs with *RUNX1-RUNX1T1* fusion.

**Figure 5:**
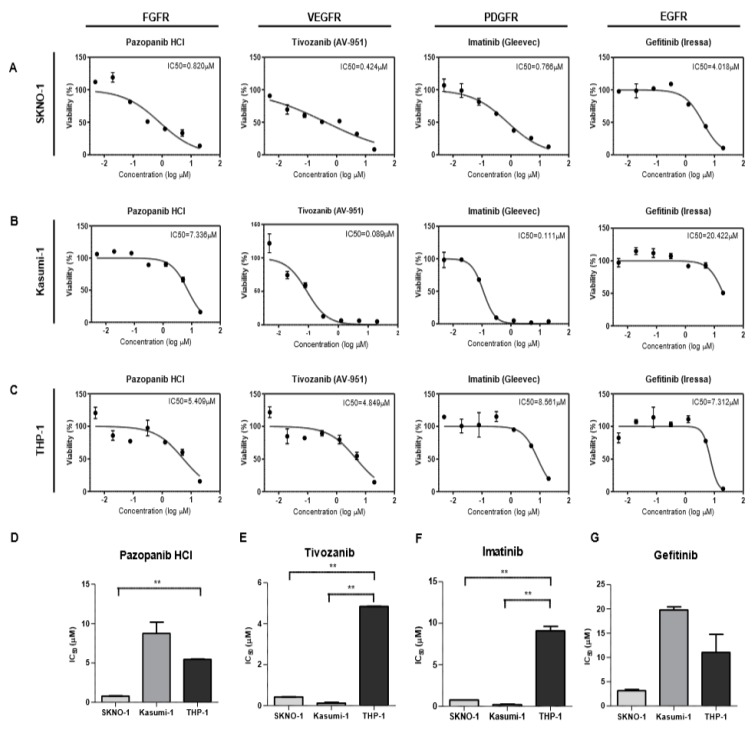
Drug response curves for leukemia cell lines. Various concentrations of pazopanib HCl, tivozanib, Imatinib, and gefitinib were treated with SKNO-1 (**A**), Kasumi-1 (**B**), and THP-1 (**C**) cells to inhibit the activities of FGFR, VEGFR, PDGFR, and EGFR, respectively. (**D**–**G**) Bar graphs compare the IC_50_ values of three cell lines for each inhibitor. ** *p* < 0.01.

When EGFRs, FGFRs, PDGFRs or VEGFRs were inhibited pharmacologically in vitro, pazopanib (for FGFRs), tivozanib (for VEGFRs), and imatinib (for PDGFRs) showed significantly lower IC_50_s in the SKNO-1 cells (Figure 5A) compared with the THP-1 cells (Figure 5C). The Kasumi-1 (Figure 5B) cells also had significantly lower IC_50_s in tivozanib and imatinib, compared with the THP-1 cells (Figure 5C). These results indicated that the survival of the SKNO-1 cells is specifically dependent on the activities of the FGFRs (Figure 5D), VEGFRs (Figure 5E), and PDGFRs (Figure 5F), while Kasumi-1 cells rely on the functions of VEGFRs (Figure 5E) and PDGFRs (Figure 5F). On the contrary, the IC_50_s of the EGFR inhibitor in the SKNO-1 (Figure 5A), Kasumi-1 (Figure 5B), and THP-1 (Figure 5C) cells did not significantly differ (Figure 5G). Moreover, the VEGR inhibitor had a lower IC_50_ than the FGFR and PDGFR inhibitors in SKNO-1 (Figure S3A) and the PDGFR and VEGFR inhibitors had lower IC_50_s than the FGFR inhibitor in Katumi-1 (Figure S3B); this is well-matched with the phosphorylation status of RTKs (Figure 4). In contrast, the effects of the FGFR, PDGFR, and VEGFR inhibitors in THP-1 did not differ compared with the EGFR inhibitors (Figure S3C) [22].

## 3. Discussion

In this study, we investigated cancer-related targetable pathways that are specifically regulated by the *RUNX1*-*RUNX1T1* fusion in AMLs. The unique aspect of the pathway analysis of this study is that the gene sets for ORA were selected based on the expression correlation with the reference gene, *RUNX1T1*. The *RUNX1T1* expression level in each RNA-seq sample might be skewed by several factors, such as its tumor purity or subclonal heterogeneity. Expression of the genes of the *RUNX1T1* downstream pathways could be affected in a similar pattern. Therefore, the gene set selection based on the expressional correlation with *RUNX1T1* has strong potential, since it offers gene selection while considering the extent of the altered gene expression in each sample.

Some cancer-related pathways identified in this study were well-matched with those of previous studies of *RUNX1-RUNX1T1* fusion-positive AMLs. For example, we identified that COX-related pathways might be activated in AMLs with the *RUNX1*-*RUNX1T1* fusion. Accordingly, it was reported that COX-2 inhibition could reduce the proliferation of *RUNX1*-*RUNX1T1* fusion-positive AML cells in vitro, and in vivo [16]. PDGF-related pathways identified in this study can explain the reason why this leukemia subtype is related to eosinophilia [23] and elevated *CD19* expression [24]. These concurrences and consistencies with the well-known features of AMLs with the *RUNC1-RUNX1T1* rearrangement might support the validity of the analysis of this study.

Besides the COX- and PDGF-related pathways, the VEGF- and FGFR1-related pathways were also predicted to be specifically activated in AMLs with the *RUNX1-RUNX1T1* fusion in this study. The signaling pathways provide novel opportunities for tailored treatments of *RUNX1-RUNX1T1* fusion positive AMLs [25,26,27].

In parallel with the in silico analysis of this study, PDGFRs, FGFRs, and VEGFRs were highly phosphorylated in two types of AML cell lines with the *RUNX1-RUNX1T1* fusion (SKNO-1 and Kastumi-1). In contrast, the phosphorylation levels were much lower in the THP1 AML cells that have no fusion. The activation status indicated that AMLs with the *RUNX1-RUNX1T1* fusion could be more sensitive to PDGFRs, FGFRs, and VEGFRs targeting agents, which were experimentally validated in this study. Although these results are in vitro validations, and not conducted studies done on patient samples, it does shed light on new possibilities for drug reposition for AML with *RUNX1-RUNX1T1.*

Combinational chemotherapy is a future direction of effective treatments for various cancer types [28]. The US Food and Drug Administration (FDA) has approved several clinical trials on combinational chemotherapy for AML patients such as cytarabine/daunorubicin and PF-04449913(Glasdegib) [29]. Our results indicate that RTK pathway-targeting agents have adverse effects on the proliferation potency of *RUNX1-RUNX1T1* fusion cell lines. It showed the possibility that use of RTK targeting agents in combination with the conventional chemotherapeutic drugs for AMLs, like Ara-C, might have better anti-tumor effects. However, it needs more preclinical study to validate which combination strategy is the best, as there are various possible combinations.

Currently, some molecular alterations are used for AML diagnosis, such as FLT3, NPM1, and CEBPA, several of which could be targeted since their protein products have enzymatic activities. In 2017, FLT3 inhibitors were approved to treat AML patients with FLT3-ITD mutations [30]. Despite these efforts, improvement of clinical outcome in AMLs is still limited to a small population of patients [31]. This is partially because many AML-specific genetic alterations cannot be targeted pharmacologically. Therefore, the identification of targetable molecules that are closely associated with AML-specific genetic alterations is important for advancement in the precision medicine of AMLs. The analysis strategy presented in this study might be helpful in the identification of treatment targets and in extending the indication of approved target therapy agents.

In this study, we elucidated gene sets, the expressions of which are specifically changed in AMLs with the *RUNX1-RUNX1T1* fusion. Fidelity of identification was upgraded by hiring the expression correlation of candidate genes with the reference gene, *RUNX1T1* in the analysis processes. Based on the gene sets, activated signaling pathways in AMLs with the *RUNX1-RUNX1T1* fusion were predicted and their functional importance in treatment was validated experimentally. This analysis process could also be adopted in the discovery of molecular targets of AMLs that have no targetable genetic alteration. Further, we expect that combination therapy using the conventional drug (Ara-c) with RTK targetable drugs shows better anti-tumor effects. Despite these encouraging results, these suggestions are required to be further investigated with clinical samples to be fully validated. With future studies possibly validating these results, it can also provoke clinical trials for personalized treatments of AMLs.

## 4. Materials and Methods

### 4.1. Data Acquisition and Inclusion Criteria

Gene level 3(RSEM) mRNA expression for The Cancer Genome Atlas (TCGA) Acute Myeloid Leukemia (AML) study was downloaded from the Broad GDAC Firehose. Clinical information, obtained from the same site, includes chromosome analysis, fluorescence in situ hybridization (FISH) results, immunophenotyping results and other clinical features. Using the results of chromosome analysis and FISH studies, we identified seven samples with *RUNX1-RUNX1T1* fusion, which were cross-checked with elevated *RUNX1T1* expression level. For the controls, 100 samples with normal karyotype were selected. After filtering 14 samples without RNA expression data, 86 samples were used as controls.

### 4.2. Over-Representation Pathway Analysis and Gene Set Enrichment Analysis

RNA expression data from each TCGA AML sample were merged into a two-dimensional matrix. Each column of the matrix represents the patient and each row represents each gene name. The RNA expression values were described as normalized read counts. To obtain the genes that are correlated in RNA expression with the reference gene (*RUNX1T1*) in 93 samples, a Pearson correlation test and Spearman correlation test were performed for each gene. Genes with R > 0.3 in both Pearson and Spearman correlation tests were selected, and 294 genes were finally selected as genes closely correlated with *RUNX1T1* in RNA expression. With the 294 genes, over-representation analysis (ORA) was performed using ConsensusPathDB (CPDB, http://consensuspathdb.org/) according to current protocols [32]. In this analysis, 4011 pathways, curated from multiple sources including INOH [33], NetPath [34], Reactome [35], HumanCyc [36], KEGG [37], Wikipathways [38], SMPDB [39], PharmGKB [40], EHMN [41] and, Signalink [42] were used.

Gene Set Enrichment Analysis (GSEA) [43] was performed to crosscheck the ORA result. The GSEA analysis software (version 2.2.3) was downloaded from the Broad Institute website (http://www.broadinstitute.org/gsea/index.jsp). The curated gene set provided by CPDB was also downloaded and modified for GSEA analysis.

### 4.3. Network Analysis for Potential Actionable Drugs and Target Genes

To analyze and visualize the relationship between therapeutic agents and their target genes, the expressions of which were specifically altered in the AMLs with *RUNX1-RUNX1T1* fusion, Cytoscape version 3.5.1 was used [44]. Knowledge-based databases including CIViC [45] and CancerSCAN [46] were used for the analysis.

### 4.4. Statistical Analysis and Visualization

To select genes that correlate well with *RUNX1T1* in RNA expression, the Pearson correlation test and Spearman correlation test were used. To visualize the RNA expression heat map with the related pathways, the R package of the ComplexHeatmap was used [47]. All statistical analysis and visualization were performed based on the open software R version 3.4.3 [48]. We applied a *p*-value of < 0.05 for statistical relevance, and a q-value of < 0.1 for false detection rate (FDR) control.

### 4.5. Cell Culture

AML cell lines with t(8;21) chromosome translocation (Kasumi-1 and SKNO-1) were used. Kasumi-1 was obtained from American Type Culture Collection (ATCC, CRL-274) and cultured in RPMI1640 media (Gibco, Waltham, MA) supplemented with 20% fetal bovine serum (FBS). SKNO-1 was obtained from the JCRB cell bank (Osaka, Japan, JCRB 1170) and cultured in RPMI1640 supplemented with 10% FBS and 10ng/mL recombinant human granulocyte-macrophage colony-stimulating factor (GM-CSF) (Peprotech, Rocky Hill, NJ). THP-1 was obtained from the Korean Cell Line Bank (Seoul, South Korea, 40202) and cultured in RPMI 1640 media supplemented with 0.05mM 2-mercaptoethanol (Gibco) and 10% FBS.

### 4.6. Receptor Tyrosine Kinase (RTK) Phosphorylation Array

Cells were cultured with their growth media in 75T flasks. Pellets were prepared upon ~80% confluence. A lysis buffer containing a protease and phosphatase inhibitor cocktail (AAH-PRTK-1, RayBiotech, GA) was used to separate the protein from the pellets. Protein concentration was determined by the bicinchoninic acid (BCA) assay (Pierce, Rockford, IL, USA). 700µg protein was reacted with a receptor tyrosine kinase (RTK) phosphorylation array (AAH-PRTK-1) according to the manufacturer’s protocol. Spots on the array were analyzed by ImageJ software(National Institutes of Health, Maryland, USA).

### 4.7. Drug Sensitivity Test

The cells were seeded in 384-well plates at a density of 500 cells per well in triplicate for each treatment. The drugs panel consisted of 61 anti-cancer agents (Selleckchem, TX, USA) targeting oncogenic signals. Two hours after plating, the cells were treated with the drugs in a seven-point serial dilution for 6 days at 37 °C in a 5% CO_2_ humidified incubator. Cell viability was analyzed using an ATP-monitoring system based on firefly luciferase (ATPLit 1step, PerkinElmer, MA, USA). Dimethyl sulfoxide (DMSO) was included as a negative control in each plate. Controls were used for the calculation of relative cell viability for each plate, and normalization was performed on a per-plate basis. Dose-response curve (DRC) fitting was performed using GraphPad Prism 5 (GraphPad) and evaluated by measuring the half maximal inhibitory concentration (IC_50_) of the DRC. After normalization, the best-fit lines were determined, and the IC_50_ value of each curve was calculated using GraphPad Prism, ignoring the regions defined by fewer than two peaks.

## Figures and Tables

**Table 1:** Clinical and pathological characteristics of *RUNX1-RUNX1T1* fusion-positive AMLs and AMLs with normal karyotype.

		RUNX1-RUNX1T1 Fusion Positive (*n* = 7)	Normal Karyotype (*n* = 86)	Statistical Difference (U Test, Fisher’s Test)
Age at Diagnosis (Mean, ^†^SD)		44.6 ± 15.9	54.8 ± 16.9	0.001
Gender (male/total)		3/7	42/86	1
Event of death		3/7	59/86	0.217
Immunophenotype	CD19	4/4	3/28	< 0.001
	CD33	3/4	72/77	0.269
	CD34	7/7	42/63	0.094
	CD45	2/2	10/10	1
	CD56	2/2	7/33	0.061
	CD117	7/7	73/73	1
	HLA-DR	6/6	44/52	0.581
	NSE	0/5	32/78	0.151
	TdT	1/2	1/4	1
Peripheral blood (median)	Blast	67.0%	71.5%	0.002
	Hb (g/dL)	9.3	9.7	0.004
	Platelet (/µL)	40K	50.8K	0.002
Bone marrow cellularity (median)		70%	88.3%	0.001
^‡^FAB classification	M0	0	5	
	M1	2	22	
	M2	5	20	
	M3	0	1	
	M4	0	21	
	M5	0	15	
	M6	0	0	
	M7	0	1	
^§^FLT3 mutation		1/7	23/68	0.675

^†^ SD: Standard deviation; ^‡^ FAB: French-American-British; ^§^ FLT3: FMS-like tyrosine kinase 3.

**Table 2:** 24 pathways that are identified in the over-representation analysis (ORA) using CPDB-providing curated pathways (*n* = 2286).

Pathway Name	Set Size	Candidates Contained	*p*-value	*q*-value	Pathway Source	Members_Input_Overlap	Potential Target Drug for Pathway
Signaling events mediated by VEGFR1 and VEGFR2	68	7 (10.3%)	0.00003	0.0161	PID	NCK2; NEDD4; PRKCD; PLCG1; VEGFA; SHB; CAV1	VEGF/VEGFR inhibitors (bevacizumab, pazopanib)
Axon guidance	459	17 (3.7%)	0.00011	0.0214	Reactome	ITGA9; ARHGEF12; NCK2; ROBO1; CRMP1; PLCG1; DUSP10; NRCAM; SHB; VEGFA; RASAL1; RHGAP39; COL6A3; LAMC1; PITPNA; NCAM1; FGFR1	
HuR stabilizes mRNA	8	3 (37.5%)	0.000121	0.0214	Reactome	TNFSF13; ELAVL1; PRKCD	
Developmental Biology	586	19 (3.3%)	0.000245	0.0325	Reactome	ITGA9; ARHGEF12; NCK2; ROBO1; CRMP1; PLCG1; NCAM1; NRCAM; SHB; VEGFA; TDGF1; RASAL1; ARHGAP39; COL6A3; LAMC1; PITPNA; DUSP10; CTNNA2; FGFR1	
Celecoxib Pathway, Pharmacodynamics	58	5 (8.6%)	0.000984	0.077	PharmGKB	CACNA2D2; PDK1; VEGFA; HPGDS; PTGIR	COX-2 inhibitors (celecoxib)
Role of second messengers in netrin-1 signaling	4	2 (50.0%)	0.00102	0.077	Reactome	PLCG1; PITPNA	
Angiogenesis overview	61	5 (8.2%)	0.00124	0.077	Wikipathways	ROBO1; PLCG1; VEGFA; SHB; FGFR1	VEGF/VEGFR inhibitors (bevacizumab, pazopanib)
Focal adhesion - Homo sapiens (human)	207	9 (4.3%)	0.0017	0.077	KEGG	ITGA9; PDGFC; ITGB4; CCND1; VEGFA; COL6A3; LAMC1; PARVG; CAV1	
Beta1 integrin cell surface interactions	66	5 (7.6%)	0.00176	0.077	PID	ITGA9; COL6A3; LAMC1; VEGFA; MDK	
S1P1 pathway	19	3 (15.8%)	0.00188	0.077	PID	PLCB2; VEGFA; PLCG1	
Cell surface interactions at the vascular wall	101	6 (6.0%)	0.00207	0.077	Reactome	SELE; SLC16A3; SELPLG; PLCG1; ESAM; CAV1	
Signaling by PDGF	301	11 (3.7%)	0.0021	0.077	Reactome	ADCY5; PDGFC; NCK2; PRKCD; DUSP10; PLCG1; RASAL1; COL6A3; CD19; NCAM1; FGFR1	PDGFR inhibitors (imatinib, ponatinib, sunitinib)
Hemostasis	493	15 (3.0%)	0.00216	0.077	Reactome	SELE; SLC16A3; DOCK6; KIF26A; PLCG1; PTGIR; MPL; VEGFA; ESAM; SELPLG; CBX5; PDE2A; PRKCD; SERPINE2; CAV1	
actions of nitric oxide in the heart	42	4 (9.5%)	0.00222	0.077	BioCarta	PLCG1; PDE2A; VEGFA; CAV1	
Thyroxine (Thyroid Hormone) Production	6	2 (33.3%)	0.00251	0.077	Wikipathways	TPO; TRH	
Extracellular matrix organization	264	10 (3.8%)	0.00256	0.077	Reactome	ITGA9; BMP4; ITGB4; HSPG2; COL14A1; FBLN2; COL6A3; LAMC1; NCAM1; FBLN5	
VEGFA-VEGFR2 Pathway	266	10 (3.8%)	0.00271	0.077	Reactome	NCK2; PRKCD; NCAM1; RASAL1; PLCG1; VEGFA; SHB; CAV1; DUSP10; FGFR1	VEGF/VEGFR inhibitors (bevacizumab, pazopanib)
Cell adhesion molecules (CAMs) - Homo sapiens (human)	142	7 (4.9%)	0.00275	0.077	KEGG	ITGA9; NRCAM; SELE; CD34; SELPLG; ESAM; NCAM1	
Signal transduction by L1	22	3 (13.6%)	0.0029	0.077	Reactome	ITGA9; NCAM1; FGFR1	
PDGFR-alpha signaling pathway	22	3 (13.6%)	0.0029	0.077	PID	PLCG1; SHB; CAV1	PDGFR inhibitors (imatinib, ponatinib, sunitinib)
Proton Pump Inhibitor Pathway, Pharmacodynamics	46	4 (8.7%)	0.00311	0.0774	PharmGKB	ADCY5; PLCB2; PLCG1; AKAP2	
Laminin interactions	23	3 (13.0%)	0.0033	0.0774	Reactome	HSPG2; ITGB4; LAMC1	
Signaling by VEGF	274	10 (3.7%)	0.00335	0.0774	Reactome	NCK2; PRKCD; NCAM1; RASAL1; PLCG1; VEGFA; SHB; CAV1; DUSP10; FGFR1	VEGF/VEGFR inhibitors (bevacizumab, pazopanib)
Phospholipase C-mediated cascade: FGFR1	49	4 (8.2%)	0.00392	0.0866	Reactome	ADCY5; PLCG1; PRKCD; FGFR1	FGFR1 inhibitors (dovitinib, ponatinib)

## Supplementary Materials

Supplementary materials can be found at https://www.mdpi.com/1422-0067/20/7/1717/s1. Figure S1. Heatmap of cancer-related and other miscellaneous genes with altered expression correlating to *RUNX1T1* expression. Figure S2. Enrichment score and ranked list metric value plot on VEGF-VEGFR, PDGF, COX, and FGFR1 related pathways. Figure S3. Bar graphs represent IC50 values of SKNO-1 (A), Kasumi-1 (B), and THP-1 (C) cells for each inhibitor. * *p* < 0.05, ** *p* < 0.01. Table S1. 293 genes whose expressions are significantly correlated with that of *RUNX1T1* genes whose expressions are significantly correlated with that of *RUNX1T1.* Table S2. Genes involved in multiple pathways and literature related to role in cancer/carcinogenesis.

## Abbreviation

AML	acute myeloid leukemia
CML	chronic myeloid leukemia
TCGA	The Cancer Genome Atlas
FISH	fluorescence in situ hybridization
ORA	over-representation analysis
CPDB	ConsensusPathDB
GSEA	Gene Set Enrichment Analysis
FDR	false detection rate
FBS	fetal bovine serum
GM-CSF	granulocyte-macrophage colony-stimulating factor
BCA	bicinchoninic acid
RTK	receptor tyrosine kinase
DMSO	Dimethyl sulfoxide
DRC	Dose-response curve
IC50	half maximal inhibitory concentration
