# Supplementary material for: Elucidation of Novel Therapeutic Targets for Acute Myeloid Leukemias with RUNX1-RUNX1T1 Fusion
Source: Int J Mol Sci. 2019 Apr 6;20(7):1717. doi: 10.3390/ijms20071717 (PMC6480444; doi:10.3390/ijms20071717)
Supplement: Supplementary file 1 [file ijms-20-01717-s001.zip › supplementary material_0325/Supplementary Figure Legends.docx]

***Supplementary Figure Legends.***

**Figure S1.** Heatmap of cancer-related and other miscellaneous genes with altered expression correlating to RUNX1T1 expression.

**Figure S2.** Enrichment score and ranked list metric value plot on VEGF-VEGFR, PDGF, COX, and FGFR1 related pathways.

**Figure S3.** Bar graphs represent IC_50_ values of SKNO-1 (A), Kasumi-1 (B), and THP-1 (C) cells for each inhibitor. *p<0.05, **p<0.01
