# Supplementary material for: Elucidation of Novel Therapeutic Targets for Acute Myeloid Leukemias with RUNX1-RUNX1T1 Fusion
Source: Int J Mol Sci. 2019 Apr 6;20(7):1717. doi: 10.3390/ijms20071717 (PMC6480444; doi:10.3390/ijms20071717)
Supplement: Supplementary file 1 [file ijms-20-01717-s001.zip › supplementary material_0325/Table S2.docx]

**Table S2**. Genes involved in multiple pathways and literature related to role in cancer/carcinogenesis.

| **Gene** | **Pathway** | **Literature regarding expression levels in cancer or role in carcinogenesis** |
| --- | --- | --- |
| **PDE2A** | VEGF, COX | Frolov et al., Mol Cancer Ther. 2003 Aug;2(8):699-709. Durand et al., J Clin Endocrinol Metab. 2011 Jul;96(7):E1206-11 Morita et al., Oncol Rep. 2013 Apr;29(4):1275-84 Shen et al., FEBS J. 2014 Aug;281(16):3609-24 Fryknas et al., J Biomol Screen. 2006 Aug;11(5):457-68 |
| **ADCY5** | FGFR1, PDGF | Liang et al., Med Oncol. 2016 Oct;33(10):111 Li et al., Biomed Res Int. 2018 Jun 20;2018:6204128 Sato et al., PLoS One. 2013;8(3):e59444 |
| **PDGFC** | FGFR1, PDGF | Bartoschek et al., Biochem Biophys Res Commun. 2018 Sep 5;503(2):984-990 Campos et al., J Steroid Biochem Mol Biol. 2013 Jan;133:12-24. Progribny et al., Toxicol Appl Pharmacol. 2007 Nov 15;225(1):61-9 |
| **DUSP10** | VEGF, PDGF | Karib et al., Med Oncol. 2013 Jun;30(2):517 Png et al., Oncogene. 2016 Jan 14;35(2):206-17 |
| **NCAM1** | VEGF, PDGF | Kok-Sin et al., Oncol Rep. 2015 Jul;34(1):22-32 Sasca et al., Blood. 2019 Feb 27. pii: blood-2018-12-889725 |
| **SHB** | VEGF, PDGF | Jamalpour et al., Tumour Biol. 2018 Apr;40(4):1010428318771472 Jamalpour et al., Tumour Biol. 2017 Oct;39(10):1010428317720643 |
| **NCK2** | VEGF, PDGF | Fanelli et al., Int J Biol Markers. 2018 Jan;33(1):124-131 Labelle-Cote et al., BMC Cancer. 2011 Oct 12;11:443 |
| **VEGFA** | VEGF, COX, FGFR1 | Liu et al., Blood. 2017 Mar 16;129(11):1491-1502 Wang et al., FEBS Lett. 2014 Nov 28;588(23):4438-47 Zaravinos et al., Oncol Rep. 2012 Oct;28(4):1159-66 |
| **CAV1** | VEGF, COX, PDGF | Jin et al., BMC Cancer. 2014 May 20;14:345 Fernandez-Rojo et al., Trends Cancer. 2016 Dec;2(12):701-705 Trimmer et al., Am J Transl Res. 2013;5(1):80-91 Tirado et al., Cancer Res. 2006 Oct 15;66(20):9937-47 |
| **FGFR1** | FGFR1, PDGF, VEGF | Guo et al., Thorac Cancer. 2017 Mar;8(2):73-79 Lo et al., J Pathol. 2015 Oct;237(2):238-48 Lee et al., Int J Oncol. 2014 Aug;45(2):641-5 Freier et al., Oral Oncol. 2007 Jan;43(1):60-6 |
| **PLCG1** | FGFR1, PDGF, VEGF, COX | Tang et al., Hepatol Res. 2019 Jan 8. doi: 10.1111/hepr.13309 |
| **PRKCD** | FGFR1, PDGF, VEGF, COX | not well specified |
| **RASAL1** | VEGF, PDGF | Liu et al., J Natl Cancer Inst. 2013 Nov 6;105(21):1617-27 |
| **PLCB2** | COX, FGFR1 | Shim et al., PLoS One. 2015 Aug 26;10(8):e0136609 |
