# Supplementary figures and images for: Elucidation of Novel Therapeutic Targets for Acute Myeloid Leukemias with RUNX1-RUNX1T1 Fusion
Source: Int J Mol Sci. 2019 Apr 6;20(7):1717. doi: 10.3390/ijms20071717 (PMC6480444; doi:10.3390/ijms20071717)

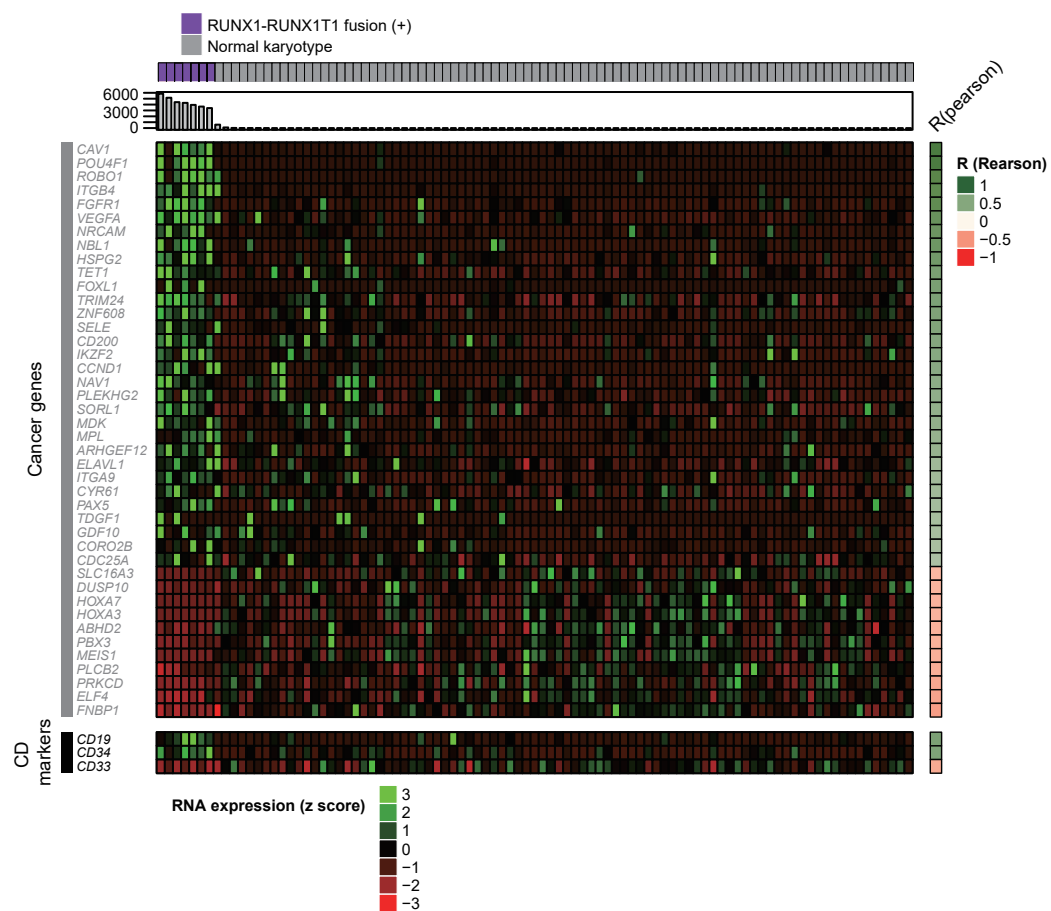

Figure S1

Supplement: Supplementary file 1 [file ijms-20-01717-s001.zip › supplementary material_0325/FigureS1.pdf]

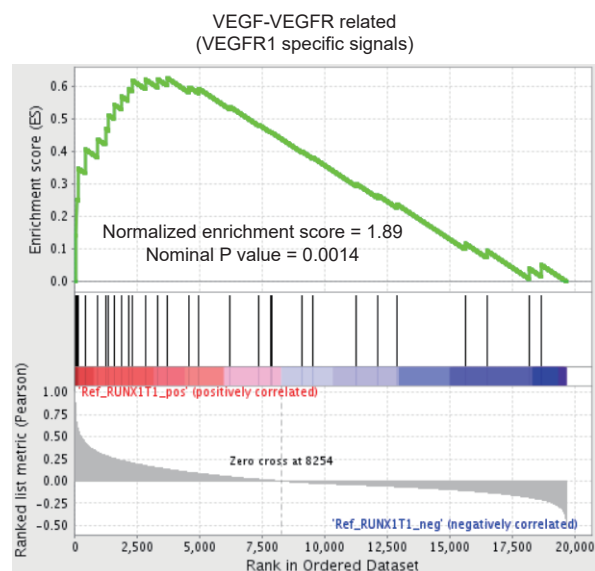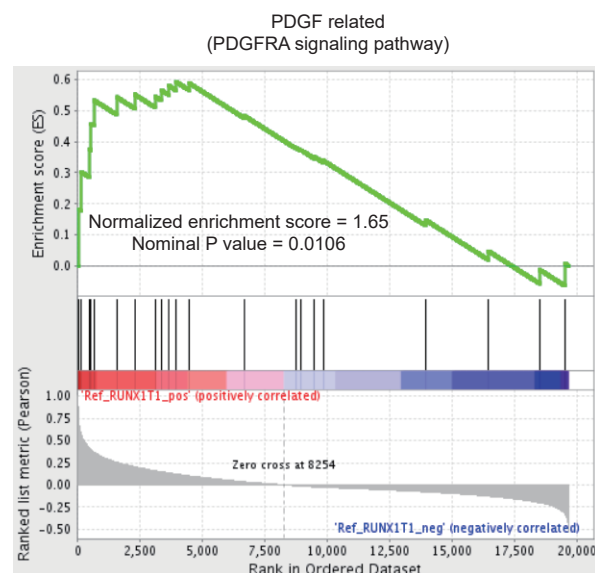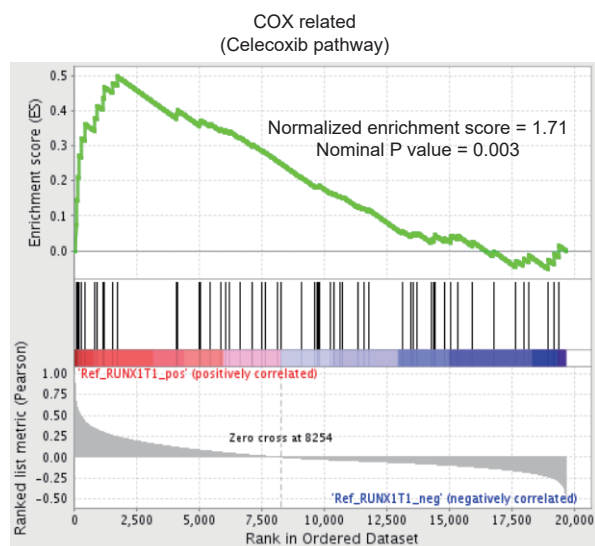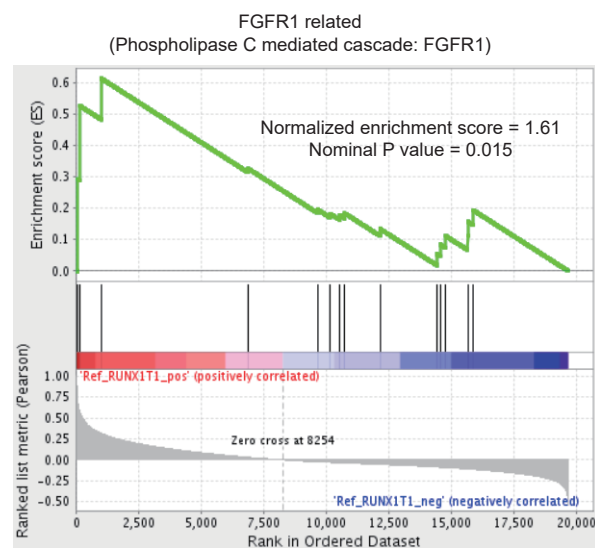

— Enrichment profile  
— Hits  
— Ranking metric scores

Figure S2

Supplement: Supplementary file 1 [file ijms-20-01717-s001.zip › supplementary material_0325/FigureS2.pdf]

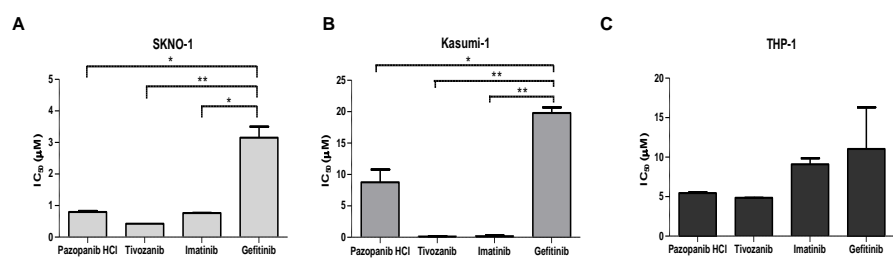

Figure S3.

Supplement: Supplementary file 1 [file ijms-20-01717-s001.zip › supplementary material_0325/FigureS3.pdf]
